# Balanced Chromosomal Rearrangements Associated with Hypoprolificacy in Australian Boars (*Sus scrofa domesticus*)

**DOI:** 10.3390/cells10082000

**Published:** 2021-08-06

**Authors:** Foyez Shams, Darryl D’Souza, Tariq Ezaz

**Affiliations:** 1 Centre for Conservation Ecology and Genomics, Institute for Applied Ecology, Faculty of Science and Technology, University of Canberra, Bruce, ACT 2617, Australia; 2 SunPork Group, Eagle Farm, QLD 4009, Australia; darryl.dsouza@sunporkfarms.com.au

**Keywords:** chromosome, karyotype, translocation, pig, reproductive performance, FISH, subtelomere

## Abstract

Balanced chromosomal rearrangements, mainly reciprocal translocations, are considered to be the causative agent of several clinical conditions in farmed pigs, resulting in hypoprolificacy and economic losses. Literature suggests that reciprocal translocations are heritable and can occur de novo. The prevalence rate of these balanced structural rearrangements of chromosomes differs from country to country and varies between 0.5% and 3.3%. The Australian pig population is descendent of a small founder population and has since been a closed genetic group since the 1980s. Hence, any incident of reciprocal translocation along with the pedigree of boars that contribute sperm for artificial insemination has the potential to have an economic consequence. To date, there has been no published account for screening of reciprocal translocation associated with hypoprolificacy in the Australian pig population. In this study, we performed standard and molecular cytogenetic analyses to identify evidence of chromosome rearrangements and their association with hypoprolificacy in a representative 94 boar samples from a commercial nucleus herd. We identified three novel rearrangements between chromosomes 5 and 14, between chromosomes 9 and 10, and between chromosomes 10 and 12. In addition, we also detected a reciprocal translocation between chromosomes 3 and 16 that has previously been detected in pig herds in France. The prevalence rate was 6.38% within the samples used in this study. All four rearrangements were found to have an association with hypoprolificacy. Further study and routine monitoring will be necessary to identify any further rearrangements that will allow breeders to prevent the propagation of reciprocal translocations from generation to generation within the Australian pig population.

## 1. Introduction

Chromosomal abnormalities, mostly balanced structural rearrangements, are known to be the source of several clinical conditions in livestock, such as congenital malfunction [1,2]. Several studies have reported a number of structural rearrangements in pigs [3,4,5,6,7] that are associated with hypoprolificacy (reduction in litter size). Reciprocal translocations (RTs) relate to the balanced chromosomal rearrangement or exchange of uneven chromosomal fragments leading to the development of genetically ‘unbalanced’ sperm. Such rearrangements result in hypoprolificacy in farmed boars. Purebred boars selected over generations based on their genetic fitness are used for breeding. Any undetected fertility issues in these boars are likely to reduce litter production within breeding populations. To avoid unwanted fertility issues, semen that is used in artificial insemination (AI) is routinely checked for usual quality assessment such as sperm concentration, morphology, and motility. However, these assessments do not detect small-scale chromosome rearrangements, such as RTs.

In pigs, more than 200 reciprocal translocations have been identified genome-wide, which cause a reduction in litter size up to 50% due to high mortality among early embryos [3,8,9]. Published data showed that approximately 50% of boars have hypoprolificacy due to reciprocal translocation with normal semen parameters [9,10]. Reciprocal translocations are heritable and can occur de novo, and do not appear to be line-specific. Propagation of RTs from generation to generation through AI of hypoprolific boars may result in significant economic loss.

Depending on the intensity of cytogenetic screening, the prevalence of RTs in boars varies between 0.5% and 1.5% [1,3,5]. Ducos et al. [3] reported that the prevalence of balanced structural rearrangement in France’s swine population is 0.47%. In another recent study, Rezaei et al. [6] reported a 0.58% prevalence in the Canadian swine population. A comparatively higher prevalence rate was estimated in the Netherlands (0.7%) and Spain (3.3%) swine populations [4,7].

The Australian pig herd has been a closed genetic population since the 1980s as a biosecurity protocol to maintain the health status of the Australian pig herd. Consequently, the Australian pig herd based on predominantly Large White, Landrace, and Duroc breeds has a relatively small founder population that may suggest a higher incidence of probable inbreeding and reduced genetic diversity within the closed herd [11]. Given the above, the prevalence rate of RTs in Australian herd and propagation of such incidents from generation to generation may be higher than previously reported. To date, there is no reported screening for reciprocal translocations or balanced chromosomal rearrangements in the Australian pig population. In this study, we performed standard and molecular cytogenetic analyses to identify evidence of chromosomal rearrangements and their association with hypoprolificacy. We tested this in a representative 94 samples from a commercial nucleus pig herd in Australia. In this genetic nucleus herd, the Large White, Landrace, and Duroc pigs are being used for breeding while Large White and Landrace are used as pure terminal sire lines.

## 2. Materials and Methods

### 2.1. Animals

A total of 94 boars were randomly selected across multiple pure terminal sire lines and included boars that had just entered service with no matings to boars with greater than 60 matings. All matings undertaken at this nucleus herd site were based on single animal semen matings. Only single sire semen matings were undertaken. All the sample collection procedures were performed by approved animal ethics protocol PIC PP 116/19.

### 2.2. Metaphase Chromosome Preparation and Karyotyping

Mitotic chromosomes were prepared from a non-synchronised culture of lymphocytes using whole blood following the protocol described by O’Connor et al. [12]. Briefly, heparinised blood samples were cultured for 72 h in PB-MAX karyotyping medium (Gibco) at 37 °C with 5% CO_2_. To arrest the cells at the metaphase stage, 0.1 µg colcemid (Gibco) was added to the 10 mL volume of culture and incubated at 37 °C for 30 min. Hypotonic treatment (0.075 M KCL, Gibco) was followed by pre-fixation and fixation in Carnoy’s solution (methanol:acetic acid 3:1). Fixed metaphase cells were dropped onto glass slides and air-dried. Cells at metaphase were stained with DAPI (4′,6-diamidino-2-phenylindole) in VECTASHIELD antifade mounting media (Vector Laboratories, Burlingame, CA, USA) prior to karyotype. All 94 boars were karyotyped to identify reciprocal translocations.

### 2.3. Fluorescence In Situ Hybridisation (FISH)

Fluorescence in situ hybridisation (FISH) was carried out using a multiprobe device, specific to porcine, developed by O’Connor et al. [12] using subtelomeric genes. The ready-to-use devices were purchased from Cytocell Ltd. (Oxford Gene Technology, Cambridge, UK). Each device contained 19 fluorescently labelled probes from distal p-arm and 19 distal q-arm of all 18 pairs of autosomes plus the X chromosome. Probes from the distal p-arm were labelled with FITC, and the q-arm were labelled with Texas Red (for details, see O’Connor et al. [12]).

FISH experiments were performed following the manufacturer’s instructions (Cytocell Ltd.). Briefly, 2 µL of cell suspension was added to each square of the glass slide, followed by washing with 2 µL of fixative (methanol:acetic acid 3:1). Air-dried cells were further washed by dipping the slide into 70% acetic acid for 5 s and 2× sodium saline citrate (SSC) for 2 min. Cells on the glass slide were dehydrated by an ethanol series (2 min each in 70%, 85%, and 100% ethanol) at room temperature. One microliter of formamide-based hybridisation buffer (Cytocell Hyb I) was pipetted onto each square of the device to resuspend the probes. The glass slide containing air-dried metaphase cells was aligned over the device containing the probes in hybridisation buffer, pressed together, and warmed on a 37 °C hotplate for 10 min. Probes and target DNA were subsequently denatured on a 75 °C hotplate for 5 min. After denaturation, the device and glass slide sandwich were placed in a dry hybridisation chamber (Cytocell Ltd.) and incubated overnight in a 37 °C water bath. Following hybridisation, slides were washed (2 min in 0.4× sodium saline citrate at 72 °C; 30 s in 2× sodium saline citrate/0.05% Tween 20 at room temperature), then counterstained using DAPI in VECTASHIELD antifade medium.

FISH images were captured using a Zeiss Axioplan epifluorescence microscope equipped with a CCD (charge-coupled device) camera (RT-Spot) (Zeiss, Oberkochen, Germany) using filters 02, 10, and 15 from the Zeiss fluorescence filter set or the Pinkel filter set (Chroma technologies, filter set 8300, Bellows Falls, VT, USA). ISIS scientific imaging software (Metasystems, Altlussheim, Germany) was used for image capture and analysis, including karyotyping.

## 3. Results

**Figure 1:**
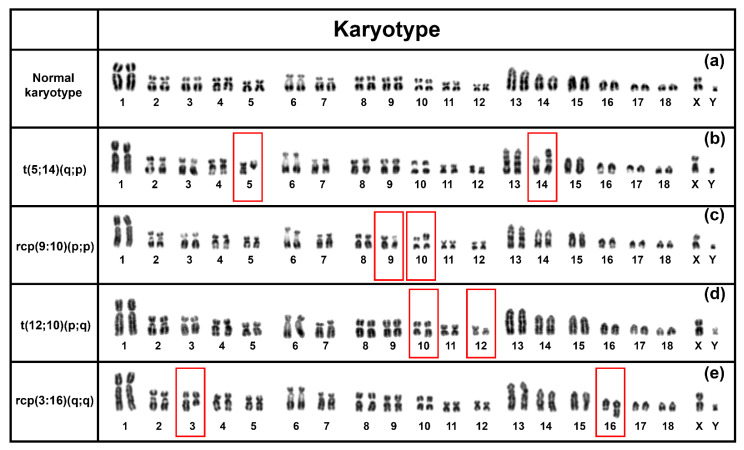
DAPI-stained karyotype of a normal boar with no chromosomal rearrangement (**a**) and four balanced chromosomal rearrangements identified in an Australian nucleus herd (**b**–**e**). Red boxes indicate the chromosomes involved in the rearrangement.

To investigate the chromosomal rearrangements in an Australian commercial nucleus herd, we performed a karyotype analysis of 94 boars. Out of 94, 6 boars were identified with 4 balanced chromosomal rearrangements (Figure 1b–e). Our analysis identified three novel balanced chromosomal rearrangements, while two of them were translocation between chromosome 5 and 14 and chromosome 12 and 10, and two reciprocal translocations between chromosome 9 and 10 and chromosomes 3 and 16 (Figure 1e).

### 3.1. t(5;14)(q;p)

Our karyotype analysis revealed a balanced translocation of the q-arm of chromosome 5 to the p-arm of chromosome 14 in three boars (Table 1). Out of three, two boars (Boar-01 and Boar-02) have been extensively used for artificial insemination (AI) and showed a marked reduction in breeding performance (Table 1). However, the third boar (Boar-03) with this translocation was a young boar that had yet to enter service. It is likely to be a common and frequent rearrangement running from generation to generation in the Australian herd given the parent-offspring relationship between Boar-01 (Father) and Boar-03 (Son), while Boar-01 and Boar-02 share a common grandfather from the dam side.

### 3.2. rcp(9:10)(p;p)

A reciprocal rearrangement between p-arm of chromosome 9 and the p-arm of chromosome 10 was identified in one boar during the screening. This rearrangement was also a novel incident. However, the current study was inconclusive to predict the association with reduced reproductive performance because of the unavailability of breeding data. The boar containing the reciprocal rearrangement was yet to enter service.

### 3.3. t(12;10)(p;q)

The karyotype analysis has indicated a second translocation within the 94 samples screened in this study. One boar had been identified with a translocation between the p-arm of chromosome 12 and the q-arm of chromosome 10. No breeding data were available from this boar.

### 3.4. rcp(3:16)(q;q)

We identified a reciprocal rearrangement between the q-arm of chromosome 3 and the q-arm of chromosome 16 (Figure 1). This reciprocal rearrangement was identified in one boar with 8.3 average PBA (pig born alive) as a reproductive performance indicator.

### 3.5. Fluorescence In Situ Hybridisation (FISH) Using Multiprobe Device

**Figure 2:**
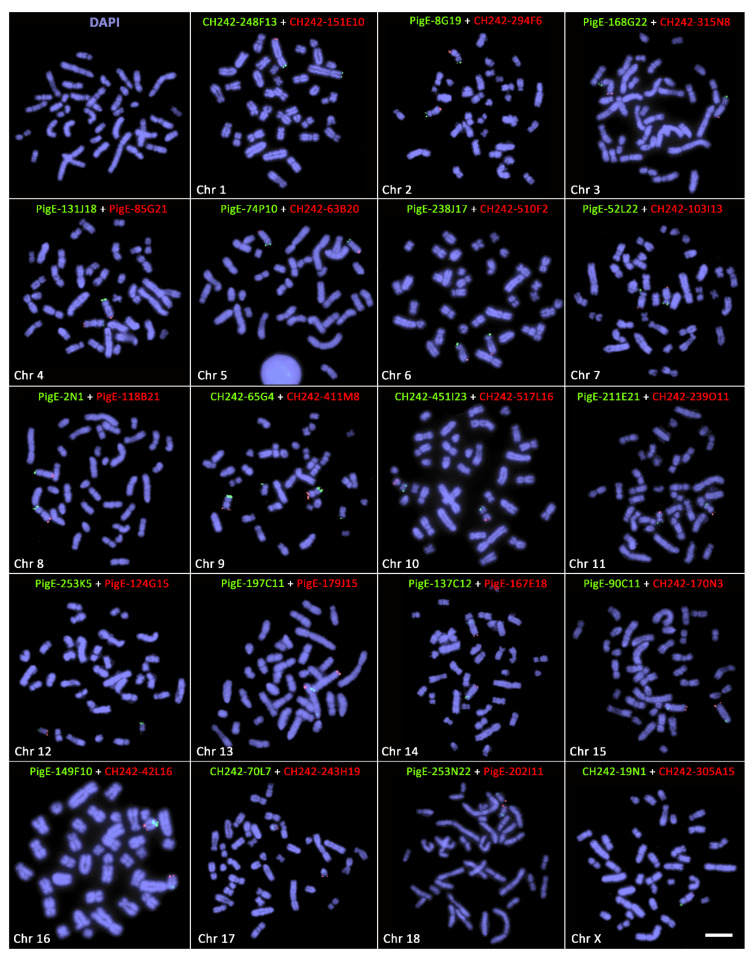
Fluorescence in situ hybridisation (FISH) signals with subtelomeric gene probes in boars with no balanced chromosomal rearrangements. Panel marked with Chr # represents the FISH signals for subtelomeric gene probes (green = specific to p-arm; red = specific to q-arm). Scale bar 10 µm.

Out of 94 boars, we screened 86 boars with chromosome-specific subtelomeric gene probes (Cytocell Ltd., Cambridge, UK) to identify small-scale rearrangements that were not detected by karyotyping as well as to validate the identified translocations and reciprocal rearrangements in boars in this study. We did not detect any new rearrangements apart from those already identified by karyotyping (Figure 2).

**Figure 3:**
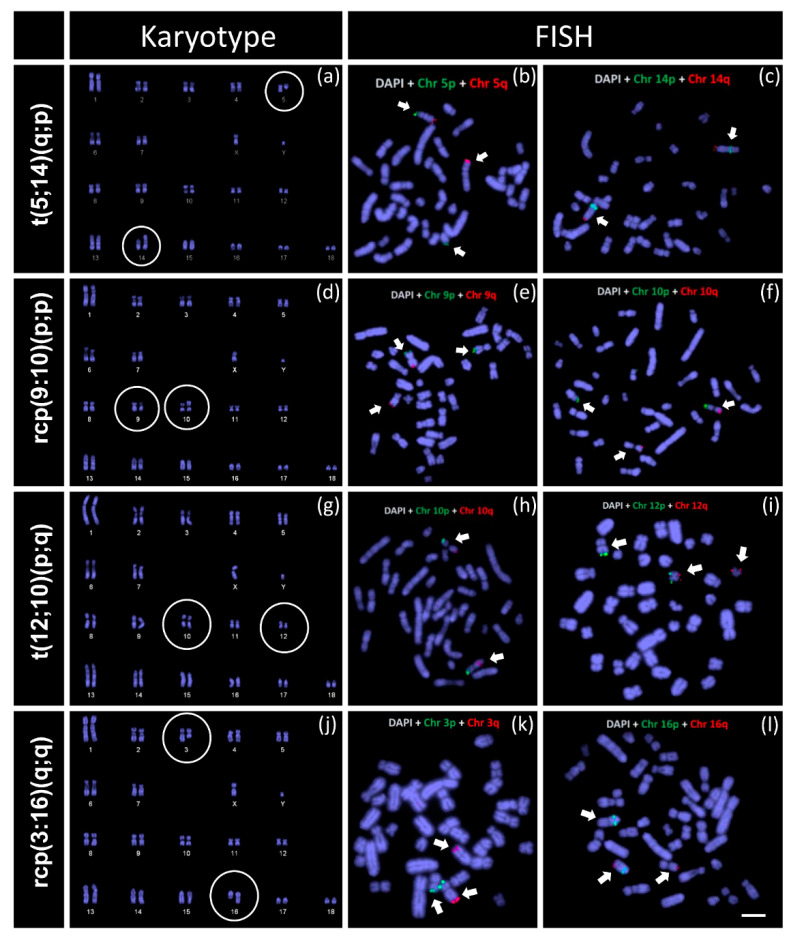
Karyotype and fluorescence in situ hybridisation (FISH) mapping of chromosome-specific subtelomeric genes in Australian boars. (**a**) DAPI-stained karyotype of t(5;14)(q;p); (**b**) FISH signals for chromosome 5-specific probes; (**c**) FISH signals for chromosome 14-specific probes; (**d**) DAPI-stained karyotype of rcp(9:10)(p;p); (**e**) FISH signals for chromosome 9-specific probes; (**f**) FISH signals for chromosome 10-specific probes; (**g**) DAPI-stained karyotype of t(10:12)(p;q); (**h**) FISH signals for chromosome 10-specific probes; (**i**) FISH signals for chromosome 12-specific probes; (**j**) DAPI-stained karyotype of rcp(3:16)(q;q); (**k**) FISH signals for chromosome 3-specific probes and (**l**) FISH signals for chromosome 16-specific probes. Scale bar 10 µm.

For all four balanced chromosomal rearrangements, we observed correct mapping of subtelomeric probes in one chromosome of the representative chromosome pair (for example, chromosome 5 while using probes that are specific to the p and q-arm of chromosome 5) while additional signals in one or two non-homologous chromosomes (Figure 3). For instance, in t(5;14)(q;p), it was evident one normal chromosome for each homologous pair chromosome 5 and chromosome 14 (green signals at the terminal position of p-arm and red signal at the terminal position of q-arm of the chromosome). However, the FISH signals for the other homolog of chromosome 5 were detected in two different chromosomes (Figure 3a–c). The hybridisation signal for the p-arm-specific probe was detected in a small acrocentric-like chromosome, while the q-arm-specific probe was detected on a large submetacentric-like chromosome. On the other hand, for chromosome 14-specific probes, the green (p-arm-specific) signal was detected in the pericentromeric region of a large submetacentric chromosome. Similarly, in t(12;10)(p;q) (Figure 3g–i), the FISH signals indicated the translocation of the p-arm of chromosome 12 to q-arm of chromosome 10 (in Figure 3h, the q-arm-specific signal was not in the terminal region of the chromosome).

In contrast to t(5;14)(q;p) and t(12;10)(p;q), the FISH signals confirmed an exchange of short segments between chromosome 9 and chromosome 10 (rcp(9:10)(p;p)) (Figure 3d–f) and chromosome 3 and chromosome 16 (rcp(3:16)(q;q)) (Figure 3j–l). Our karyotype analysis was successful in identifying all the rearrangements. However, to find out which chromosomes were involved in rearrangements and particularly for rcp(9:10)(p;p) and rcp(3:16)(q;q), where rearrangements as picked up by karyotyping were too subtle to be 100% sure.

## 4. Discussion

Our cytogenetic screening in purebred boars in a commercial nucleus pig herd from Australia identified a comparatively higher (6.38%) prevalence of reciprocal translocations. This prevalence rate is similar to several other published reports in other commercial herds spanning multiple countries, such as France (0.47% in 7700 young boar), Canada (0.58% in 5481 boars), Spain (3.8% in 849 pigs) [3,6,7]. Our data also suggest an association of reduced reproductive success in the hypoprolific boars with reciprocal chromosome translocations (Table 1).

The comparison among four chromosomal rearrangements identified in this study indicates a higher incidence rate of translocation between chromosome 5 and chromosome 14 (t(5;14)(q;p) in three boars). We also identified three novel chromosomal rearrangements in the Australian herd (t(5;14)(q;p), rcp(9:10)(p;p), and t(12;10)(p;q)). A similar translocation of chromosome 3 to chromosome 16 (rcp(3:16)(q;q)) was previously reported in Ducos et al. [3]. However, further analysis, such as mapping genes (e.g., BAC clones) bordering the breakpoints, will be required to characterise the rearrangements that we identified and that reported by Ducos et al. [3] are identical.

The mechanisms of reciprocal translocations are yet to be fully understood. However, recent studies suggest that the likelihood of translocations in the mammalian genome is associated with multiple chromosome features, such as physical length of chromosomes, frequency of translocation breakpoints, presence of common fragile sites, and chromatin density (e.g., heterochromatin or euchromatin) [8,10,13,14,15,16,17,18,19]. In a recent study, Donaldson et al. [20] reported a significant association of translocation frequency to the length of chromosome arms where larger chromosome arms tend to possess more translocation breakpoints. We also observed similar patterns among the RTs identified in the current study. About 50% of identified RTs (2 out of 4) were in non-metacentric chromosomes, and all of them involved the long arm of the chromosome, such as the q-arm of chromosome 5 in t(5;14)(q;p) and q arms of chromosome 3 and chromosome 16 in rcp(3:16)(q;q) (Figure 3).

One limitation of this study is that the breakpoint sites of the identified rearrangements are not detectable using the fluorescence in situ hybridisation (FISH) experiment of subtelomeric probes. However, considering the distribution of translocation breakpoints and common fragile sites in pig chromosomes reported by Donaldson et al. [20], we presume that all the rearrangements are associated with either a translocation breakpoint or a common fragile site (Figure S1). For instance, the q arms of chromosome 3, chromosome 5, and chromosome 16 possess five translocation breakpoints each. We identified four boars containing RTs involving these chromosomes. The translocation breakpoints of metacentric chromosome 10 and chromosome 12 are likely to be associated with common fragile sites in the p-arm of both chromosomes (Figure S1). The translocation breakpoint of chromosome 9 in rcp(9:10)(p;p) could be one of the two translocation breakpoints, and potentially the most frequent is 9p24. Although these are assumptions based on empirical data, we suggest further analysis with chromosome banding to pinpoint the breakpoints of RTs identified in the Australian pig population.

The prolificacy data indicates that three (Boar-01, Boar-02, and Boar-06) out of six boars with chromosomal abnormalities were used in breeding programs, and all three showed a marked reduction in litter size between 25% and 57% (average PBA 11.2 based on breeding performance of contemporary boars of this study). This could be a sole effect of the translocation or a combination of the translocation and other unknown factors that resulted in reduction in litter size and sterility. Sterility in boars that carry a chromosomal rearrangement, especially autosomal rearrangements, has previously been reported in an experimental farm-bred boar in Siberia by Astachova et al. [21].

We detected about 49% (Boar-01) and 57% (Boar-02) reduction in litter sizes in two boars that carry a translocation between chromosome 5 and 14 (t(5;14)(q;p)). Although this study does not provide conclusive evidence, our prediction is that the translocation between chromosome 5 and chromosome 14 has been inherited in multiple generations. Particularly considering the family relationship among all three boars (Boar-01, Boar-02, and Boar-03) that carry the translocation. In the pedigree, Boar-01 and Boar-03 are father and son, while Boar-01 and Boar-02 share a common grandfather from the dam side.

We detected comparatively higher reproductive success in the hypoprolific boar carrying the reciprocal translocation between chromosome 3 and chromosome 16 (25% reduction in litter size). A similar reciprocal translocation was previously reported in the French swine population by Ducos et al. [3], resulting in hypoprolificacy and production of several malformed piglets; however, rates of incidences of RTs are not known. The karyotype analysis of the offspring performed by Ducos et al. [3] identified propagation of the same translocation, the incidence of partial trisomy of chromosome 3, as well as partial monosomy of chromosome 16.

Despite a large number (more than 140) of chromosomal rearrangements reported from France’s swine population in the early 2000s [3], novel rearrangements are still being reported from different swine populations all over the world [4,6,7]. The present study identified three novel chromosomal rearrangements that can be characterised as the causative agent of hypoprolificacy as well as probable sterility. Further studies through a continuous cytogenetic screening will be necessary to advise the probable number of balanced chromosomal rearrangements in the Australian pig herd.

The semen quality assessment methods used in boar studs in Australia do not detect chromosomal rearrangements, such as RTs. The results reported in this study have identified a high incidence (6.38%) of these rearrangements in a commercial Australian boar population and appear to be higher than in other pig populations (<1–4%) [3,6,7]. A minimum of 30 matings across multiple sows is a common practice in genetic nucleus herds to ensure no maternal confounding factors are affecting terminal sire performance. The litter size data for the RT boars reported in this study had a minimum of 30 matings. Hence, we are confident that there are no maternal factors confounding these data.

As mentioned previously, the higher RTs incidence in Australia could be attributed to the Australian pig herd being a closed genetic herd. It is likely that these results are also applicable to other pig herds in Australia. This is a possibility, given that previous studies have identified that most herds in Australia share common ancestry [22]. Another possible reason for the high prevalence rate of reciprocal translocation in Australia could be the low number of boars screened in this study compared to other European countries.

## 5. Conclusions

The cytogenetic screening for balanced chromosomal rearrangements in a commercial Australian genetic nucleus herd indicates a high rate of prevalence compared to other European countries. This is the first report for the screening of hypoprolific boars where we report four reciprocal translocations associated with hypoprolificacy in the Australian swine herd, of which three RTs are novel and likely to have originated within Australian herds. Our detection of such a high prevalence rate RTs in the Australian swine population highlights risks of breeding using a close genetic herd that is likely to lead to inbreeding depression. With these limitations, it is, therefore, necessary to implement routine cytogenetic screening in all Australian herds, in particular to those larger farms that supply genetics (semen) for smaller breeders.

## Figures and Tables

**Table 1:** Phenotypic performance data (total pigs born and pigs born alive) for boars identified with reciprocal translocations. ^A^ Boar had just entered the breeding program, and semen had not yet been used for on-test AI mating.

Boar ID	Karyotype	Rearrangement	Total Pigs Born	Total Pigs Born Alive
Boar-01	Translocation	t(5;14)(q;p)	5.9	5.6
Boar-02	Translocation		5.5	4.8
Boar-03	Translocation		No matings ^A^	
Boar-04	Translocation	rcp(9:10)(p;p)	No matings ^A^	
Boar-05	Translocation	t(12;10)(p;q)	No matings ^A^	
Boar-06	Translocation	rcp(3:16)(q;q)	9.2	8.3
Average for all normal boars	Normal	Normal	12.9	11.2

## Data Availability

All data is contained within the manuscript.

## Supplementary Materials

The supplementary figure is available online at https://www.mdpi.com/article/10.3390/cells10082000/s1, Figure S1: The comparison of chromosomal rearrangements evident in the current study and translocation breakpoints and common fragile sites in the pig karyotype presented by Donaldson et al. [20].

## References

[1] Ducos A., Revay T., Kovacs A., Hidas A., Pinton A., Bonnet-Garnier A., Molteni L., Slota E., Switonski M., Arruga M.V. (2008). Cytogenetic screening of livestock populations in Europe: An overview. Cytogenet. Genome Res..

[2] Yimer N., Rosnina Y. (2014). Chromosomal Anomalies and Infertility in Farm Animals: A Review. Pertanika J. Trop. Agric. Sci..

[3] Ducos A., Berland H.-M., Bonnet N., Calgaro A., Billoux S., Mary N., Garnier-Bonnet A., Darré R., Pinton A. (2007). Chromosomal control of pig populations in France: 2002–2006 survey. Genet. Sel. Evol..

[4] Feitsma H. (2009). Artificial insemination in pigs, research and developments in The Netherlands, a review. Acta Sci. Vet..

[5] Quach A.T., Revay T., Villagomez D.A.F., Macedo M.P., Sullivan A., Maignel L., Wyss S., Sullivan B., King W.A. (2016). Prevalence and consequences of chromosomal abnormalities in Canadian commercial swine herds. Genet. Sel. Evol..

[6] Rezaei S., Donaldson B., Villagomez D.A.F., Revay T., Mary N., Grossi D.A., King W.A. (2020). Routine Karyotyping Reveals Frequent Mosaic Reciprocal Chromosome Translocations in Swine: Prevalence, Pedigree, and Litter Size. Sci. Rep..

[7] Sánchez-Sánchez R., Gómez-Fidalgo E., Pérez-Garnelo S., Martín-Lluch M., De la Cruz-Vigo P. (2019). Prevalence of chromosomal aberrations in breeding pigs in Spain. Reprod. Domest. Anim..

[8] Rothschild M.F., Ruvinsky A. (2011). The Genetics of the Pig.

[9] Rodríguez A., Sanz E., De Mercado E., Gómez E., Martín M., Carrascosa C., Gomez-Fidalgo E., Villagómez D.A.F., Sánchez-Sánchez R. (2010). Reproductive consequences of a reciprocal chromosomal translocation in two Duroc boars used to provide semen for artificial insemination. Theriogenology.

[10] Donaldson B., Villagomez D.A.F., King W.A. (2021). Classical, Molecular, and Genomic Cytogenetics of the Pig, a Clinical Perspective. Animals.

[11] Bunter K.L., Hermesch S. (2017). What does the ‘closed herd’ really mean for Australian breeding companies and their customers?. Anim. Prod. Sci..

[12] O’Connor R., Fonseka G., Frodsham R., Archibald A.L., Lawrie M., Walling G.A., Griffin D.K. (2017). Isolation of subtelomeric sequences of porcine chromosomes for translocation screening reveals errors in the pig genome assembly. Anim. Genet..

[13] Basrur P., Stranzinger G. (2008). Veterinary cytogenetics: Past and perspective. Cytogenet. Genome Res..

[14] Warburton D. (1991). De novo balanced chromosome rearrangements and extra marker chromosomes identified at prenatal diagnosis: Clinical significance and distribution of breakpoints. Am. J. Hum. Genet..

[15] Bickmore W.A., Teague P. (2002). Influences of chromosome size, gene density and nuclear position on the frequency of constitutional translocations in the human population. Chromosome Res..

[16] Yu C., Borgaonkar D., Bolling D. (1978). Break points in human chromosomes. Hum. Hered..

[17] Aurias A., Prieur M., Dutrillaux B., Lejeune J. (1978). Systematic analysis of 95 reciprocal translocations of autosomes. Hum. Genet..

[18] Lin C.-Y., Shukla A., Grady J.P., Fink J.L., Dray E., Duijf P.H. (2018). Translocation breakpoints preferentially occur in euchromatin and acrocentric chromosomes. Cancers.

[19] Rønne M. (1995). Localization of fragile sites in the karyotype of Sus scrofa domestica: Present status. Hereditas.

[20] Donaldson B., Villagomez D.A., Revay T., Rezaei S., King W.A. (2019). Non-random distribution of reciprocal translocation breakpoints in the pig genome. Genes.

[21] Astachova N.M., Vysotskaya L.V., Graphodatsky A.S. (1991). Detailed analysis of a new translocation in pig. Genet. Sel. Evol..

[22] John J.C.S., Tsai T.-S. (2018). The association of mitochondrial DNA haplotypes and phenotypic traits in pigs. BMC Genet..

