# Supplementary material for: Balanced Chromosomal Rearrangements Associated with Hypoprolificacy in Australian Boars (Sus scrofa domesticus)
Source: Cells. 2021 Aug 6;10(8):2000. doi: 10.3390/cells10082000 (PMC8394656; doi:10.3390/cells10082000)
Supplement: Supplementary file 1 [file cells-10-02000-s001.zip › cells-1313698-supplementary.pdf]

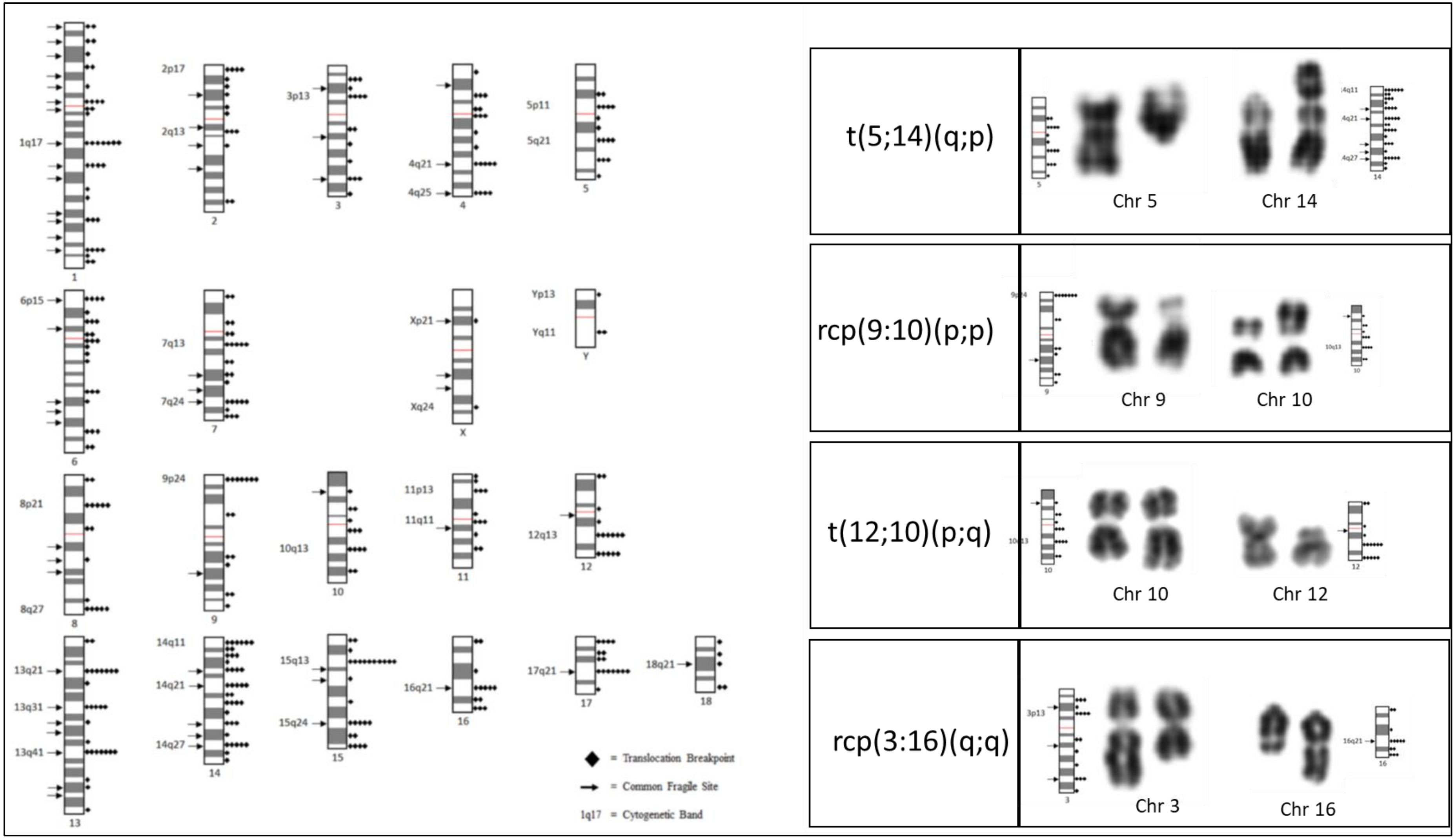

Figure S1: The comparison of chromosomal rearrangements evident in the current study and translocation breakpoints and common fragile sites in the pig karyotype presented by Donaldson et al. [20]
